# Effects of the Paleo diet on resting metabolic rate in handball players

**DOI:** 10.1038/s41598-026-58374-5

**Published:** 2026-06-16

**Authors:** Aleksandra Pięta, Paulina Mazur-Kurach, Marcin Maciejczyk, Barbara Frączek

**Affiliations:** 1 Department of Sports Medicine and Human Nutrition, Institute of Biomedical Sciences, University of Physical Culture, Krakow, Poland; 2 Department of Physiology and Biochemistry, Institute of Biomedical Sciences, University of Physical Culture, Krakow, Poland

**Keywords:** Paleolithic diet, Resting metabolic rate, Substrate utilization, Moderate-carbohydrates diet, Athletes, Nutrition, Metabolism

## Abstract

The Paleolithic diet (PD) has positive effects on health status and body composition. The Paleo diet has been also used to assess the effects on handball players performance but the association of adherence to PD and resting metabolic rate (RMR) has not been investigated especially in this group. This study aimed to impact of the moderate-carbohydrate diet on RMR among professional handball players. Twenty-five handball players were assigned into two groups: 14 in the experimental group (the Paleo diet; PD) and 11 in the control group (rational diet; CD) for 8-weeks of normoenergetic nutritional intervention. Resting metabolic rate was measured by indirect calorimetry using a Cortex MetaLyzer 3R ergospirometer (Germany), using the breath-by-breath method. Oxygen uptake (VO_2_), carbon dioxide production (VCO_2_), respiratory quotient (RQ), RMR, and substrate utilization (carbohydrate, fat, protein) and energy expenditure from each substrate were measured during the measurement. There were no differences in RMR parameters, as well as VO_2_ (L/min), VCO_2_ (L/min), RQ, substrate utilization (g/day): CHO, PRO, FAT, and EE (kcal/hour) from each substrate between PD and CD groups. Eight weeks of a normoenergetic Paleo diet did not affect resting metabolic rate.

**Supplementary Information:**

The online version contains supplementary material available at https://doi.org/10.1038/s41598-026-58374-5.

## Introduction

The measurement of metabolic rate in an outpatient setting in a fasted and rested state, is known as resting metabolic rate (RMR)^1^. Resting metabolic rate is typically higher than basal metabolic rate (BMR). Basal metabolic rate represents the minimal energy cost of living and makes up one of the components of total daily energy requirements alongside the thermic effect of food, exercise activity thermogenesis, and non-exercise activity thermogenesis^2^. In these two rates, similar results are seen when there is an adequate period of rest prior to the RMR measurement^3^. While an athlete’s RMR can be estimated from predictive equations laboratory testing using indirect calorimetry is recommended^4^. RMR/BMR is affected by age, physical activity, and lean body mass. Long-term effects of physical activity, particularly resistance training, may result in modest increases in RMR due to an increase in lean muscle mass, which is correlated with RMR^5,6^. Many other factors, like anxiety, diurnal variation, elevated post-exercise oxygen consumption, stimulants, and pharmaceuticals can also affect the resting metabolic rate^7,8^. Knowledge of an athlete’s RMR has valuable information on energy requirements^9^, which can help guide support staff who give nutrition advice and plan the diet of an athlete. Changes in resting metabolism may explain the potential reduction effects of some diets, especially recently fashionable alternative strategies. Of note, impact of alternative diets with different amount of macronutrients in professional sport are also insufficiently researched. The goal of experiments undertaken by physiologists and nutritionists is to evaluate the relationship between the type of diet and the RMR, as well as utilization of energy substrates in rest and during exercise^10–14^. The mechanisms of dietary effects on metabolism are still ambiguous.

There are many scientific articles that evaluate effects of Paleo diet (PD) on health status in people with diseases, such as blood lipid disorder^15^, overweight or obesity^16,17^, diabetes^18–20^, and metabolic syndrome^21^, and even on healthy, inactive adults^22^. Most, albeit not all, studies suggest that a PD has positive effects on body composition^22–24^. The Paleo diet has been also used to assess the impact on athletes aerobic and anaerobic performance^25^. Paleolithic nutrition consists mainly of grass-fed and pasture-raised meats, vegetables, fruits and nuts, excludes grains, legumes and dairy products plus limits refined sugars, starches, processed foods, and some oils. On average, the authors estimate the following ratio of macronutrients: 35% energy from fats, 35% from carbohydrates, and 30% from protein (although no specific amount is the goal)^21,26^. In contrast, high-carbohydrate diets are comparable to Western diets defined as a CHO proportion of total energy intake exceeding 45%. Currently, researchers evaluating the nutritional value of PD classify it as a moderate-carbohydrate diet^27^. Reduced-carbohydrate dietary strategies are of high interest to athletes^28^. The answer to the question of what effects a PD has on energy metabolism in professional athletes remains unclear. It is still puzzling how a moderate-carbohydrate diet can affect resting metabolism and macronutrient utilization in athletes. It is known that a decrease in RMR is a well-known consequence of a weight loss diet but in overweight and obese people. This phenomenon is commonly associated with a reduction in fat-free mass (FFM)^29,30^. Weight loss is accompanied by decreased leptin secretion, and changes in leptin levels have been shown to predict alterations in RMR. Recent evidence suggests that macronutrient composition may influence leptin dynamics and metabolic adaptations, particularly under energy-restricted conditions^31^. Therefore, in accordance with the existing scientific evidence, we chose a normoenergetic intervention with an alternative macronutrient distribution. In our previous study, no changes in leptin concentration were observed following a normoenergetic Paleo diet (PD)^23^.

Considering the objections to the use of PD, we wanted to assess the impact of an eight-week PD on resting metabolic rate and macronutrients utilization of professional handball players.

## Materials and methods

The sample size was determined prior to the start of the study. G*Power software version 3.1.9.7 (Germany) was used to calculate the sample size. The following data were entered into the software: test family = f tests; statistical test = ANOVA with repeated measures, within-between interaction; type of power analysis = calculation of the required sample size-with assumed α, power, and effect size. The parameters entered into the software were as follows: effect size f: 0.25; error probability α: 0.05; power: 0.80; number of groups: 2; number of measurements: 3; correlation between measurements: 0.5; non-sphericity correction: 1.0). The required total sample size was 28 participants. Due to possible dropouts from the study, 32 participants (16 in each group) were recruited. Five participants dropped out of the experiment in the first research series as they failed to maintain the nutritional regime, regardless of the diet they were to use, despite the high taste preference for the diet or a rational and expressed willingness to take up the diet indicated in the questionnaire. Two athletes ultimately failed to meet the inclusion criterion for undertaking physical activity for all subjects. The participants provided their written informed consent for voluntary participation in the trial. The inclusion criteria in the study group were age 18–35 years, at least 5 years of participation in competitive sports, undertaking regular physical activity at least 5 times a week for more than one hour per training session, and participation in national and/or international competitions.

### Characteristics of participants

The research group consisted of 25 male professional handball players: 14 in the experimental group—the Paleo diet (PD) and 11 in the control group—rational diet (control diet, CD). Handball players participating in the study were members of one team—the 2nd league team from Poland. The research was undertaken during the pre-compepition period. Players received an average of 6 discipline-specific training units per week, lasting an average of 2 h/day. Training loads were the same for everyone (featured in supplement: a detailed description of the training sessions, including their duration, exercises, and number of repetitions, Table 1a). Comparing the groups eating the PD and the CD, no differences were found in the distributions of basic data from age and anthropometric measurements (Table 2). Before starting the experiment and after the 4th, and 8th week, the body composition was measured using dual energy X-ray absorptiometry (DXA) using the Lunar iDXA instrument. Fat mass (FM) [% and kg], fat free mass (FFM) [% and kg], were analysed in this study.

### Dietary intervention

At the begining the experiment, the players performed 10-day monitoring of total energy expenditure (on 8 training days and 2 non-training days) using a heart rate monitor (Polar M400/RS400, Polar, Finland). Based on the individual energy and nutrient demand, body mass the all-day food rations were prepared with the use of the Aliant Dietetic Calculator 4.10.14 (Cambridge Diagnostics, Poland) considering the normoenergetic model of the diet. The athletes were randomly assigned to two dietary groups in a two-arm randomized controlled trial using blocked randomization (n = 32). An eight weekly food rations (ultimately 784 days for all subjects in the PD and 616 in the CD group) were created for each athlete, then prepared and delivered by the catering company. During the experiment, the athletes did not take any supplements influencing the resting and exercise metabolism. These two diets differed significantly in terms of the supply of all macronutrients [g, g/kg BM and %] except the protein supply in g/kg BM and fiber (Table 1).

**Table 1:** Macronutrients in both groups.

Macronutrient	Group	Mean ± SD	t-test *p*
Carbohydrate [g/kg BM]	PD	3.30 ± 0.79	< *0.001*
	CD	5.25 ± 0.74	
Protein [g/kg BM]	PD	2.38 ± 0.33	*0.069*
	CD	2.13 ± 0.30	
Fat [g/kg BM]	PD	2.10 ± 0.40	< *0.001*
	CD	1.48 ± 0.40	
Carbohydrates [%ED]	PD	30.29 ± 7.06	< *0.001*
	CD	48.69 ± 7.01	
Protein [%ED]	PD	23.18 ± 1.26	< *0.001*
	CD	20.07 ± 1.22	
Fat [%ED]	PD	46.53 ± 8.22	< *0.001*
	CD	31.23 ± 5.94	
Fiber [g/day]	PD	57.35 ± 8.89	0.488
	CD	59.75 ± 7.89	

*PD* Paleo diet, *CD* control diet, *BM* body mass, *ED* energy demand, *SD* standard deviation.

### Measurement of resting metabolic rate

Resting metabolic rate was measured: baseline-on the first day of the experiment, after 4 weeks and after 8 weeks of nutritional intervention in the morning after 12-h overnight fasting, always at the same time of day, between 6:00 a.m. and 10:00 a.m.^7^, also showed that repeated morning measurements of RMR are stable and highly correlated and day-to-day measurements of RMR are not significantly different ^8^. Prior to the first RMR measurement, participants were instructed on how to prepare for the measurement, i.e. avoid exercise for 3 days before the scheduled measurement, be properly hydrated, and not use any stimulants before the study (nicotine, caffeine). Athletes were instructed not to fall asleep during the measurement, and this was verbally confirmed following the measurement. The room was temperature controlled, and athletes had access to a blanket during the measurement so that they were at a comfortable temperature. RMR was measured in the supine position in an air-conditioned laboratory, at a constant temperature of 21 °C, after a prior rest of about 15 min in the supine position. Resting metabolic rate, was measured by indirect calorimetry using a MetaLyzer 3R metabolic cart (Cortex, Germany), using the breath-by-breath method. The size of the Hans Rudolph face mask was individually selected for each participant, and mask tightness was checked after placement to ensure proper sealing.The metabolic cart was calibrated each time according to the manufacturer’s requirements (gas and volume calibration). Volume calibration was performed using a 3-L calibration syringe, and gas calibration was conducted using reference gases (O_2_: 15%, CO_2_:5%) as well as ambient air (ATPS conditions). After calibration, the participant was connected to the metabolic cart and the measurement was initiated. Steady state was defined as fluctuations not exceeding predefined limits for oxygen consumption (VO_2_): 10%, VCO2:6% and RQ:3%. Data were averaged over 60-s intervals, and the minimum duration required to achieve steady state was 5 min. Oxygen uptake (VO_2_), carbon dioxide production (VCO_2_), respiratory quotient (RQ), RMR, and substrate utilization (carbohydrate [CHO], fat [FAT], protein [PRO]) and energy expenditure (EE) from each substrate were measured during the measurement. Resting metabolism was expressed absolutely (kcal/day) and relatively to body mass (kcal/kg/day) and to body surface area (RMR/BSA) (kcal/m^2^/day). The averages from steady state were used to calculate REE using the Weir formula without using urinary urea nitrogen^32^. Calculation of substrates was based on assumed share of protein utilization in energy expenditure and was set at 5%. All calculations were performed using dedicated metabolic rate measurement software provided by manufacturer of the metabolic cart (Cortex, Germany).

### Statistical analysis

The PQStat statistical package, version 1.8.0.338 was used for the statistical analysis of the collected results and their interpretation. The basic characteristics of the examined variables were calculated, i.e.: mean and standard deviation (SD). Data distribution was checked using the Shapiro–Wilk test. Homogeneity of variance within the groups was tested via Levene’s test Analysis of variance (ANOVA) with repeated measures or one-way ANOVA was used to analyze the data obtained. Post-hoc analysis was performed using the Bonferroni correction. As the macronutrient intake variables were normally distributed according to the Shapiro–Wilk test, comparisons between the PD and CD groups were performed using an independent-samples t-test. Since FFM is a major determinant of RMR, in the case of RMR, repeated measures ANCOVA were performed with diet and time as factors and FFM as a covariate. ANCOVA and then post-hoc analysis were performed using Statistica software (StatSoft, USA).

## Results

There were no significant intergroup changes in body mass, BMI, FFM, FM and resting metabolic rate parameters. There were also no differences in VO_2_ (L/min), VCO_2_ (L/min), RQ, substrate utilization (g/day): CHO, PRO, FAT, and EE (kcal/hour) from CHO, PRO and FAT between PD and CD groups (Table 2).

**Table 2:** Basic anthropometric measurements, resting metabolic rate and substrate use in PD and CD on different measurement points.

Variable	Group	Baseline (T1) Mean ± SD	4 weeks (T2) Mean ± SD	8 weeks (T3) Mean ± SD	Effect: group F (*p*)	Effect: time F (*p*)	Interaction group x time F (*p*)	Effect: FFM* F (*p*)	Interaction* time x FFM F (*p*)	Time change: pre vs. post (post hoc) *p*
Age (years)	PD	21.07 ± 2.09	–	–	(0.05)					
	CD	23.09 ± 2.57	–	–						
BH (cm)	PD	185.93 ± 7.49	–	–	(0.60)					
	CD	184.36 ± 6.51	–	–						
BM (kg)	PD	89.68 ± 10.77	88.20 ± 10.93	87.66 ± 10.37	0.21 (0.65)	14.92 (< 0.01)	0.75 (0.48)			T1 vs T2 < 0.01 T2 vs T3 0.18 T1 vs T3 < 0.01
	CD	86.87 ± 14.05	86.05 ± 13.95	85.53 ± 13.59						
BMI (kg/m^2^)	PD	25.85 ± 1.77	25.41 ± 1.68	25.27 ± 1.69	0.07 (0.79)	15.11 (< 0.01)	0.76 (0.47)			T1 vs T2 < 0.01 T2 vs T3 0.21 T1 vs T3 < 0.01
	CD	25.21 ± 3.00	25.21 ± 3.15	25.06 ± 3.00						
FFM [kg]	PD	70.30 ± 8.73	70.69 ± 8.85	70.65 ± 8.51	0.01 (0.90)	0.49 (0.61)	0.29 (0.75)			
	CD	71.14 ± 5.391	71.05 ± 5.55	71.16 ± 6.14						
FM [kg]	PD	19.10 ± 9.12	17.04 ± 8.07	15.96 ± 7.76	0.21 (0.66)	34.33 (< 0.01)	0.03 (0.98)			T1 vs T2 < 0.01 T2 vs T3 < 0.01 T1 vs T3 < 0.01
	CD	18.79 ± 6.79	16.59 ± 6.07	14.53 ± 5.20						
VO_2_ (L/min)	PD	0.31 ± 0.04	0.31 ± 0.05	0.32 ± 0.05	0.28 (0.60)	0.60 (0.55)	0.18 (0.84)			
	CD	0.32 ± 0.04	0.31 ± 0.04	0.32 ± 0.03						
VCO_2_ (L/min)	PD	0.24 ± 0.03	0.24 ± 0.04	0.26 ± 0.03	0.13 (0.72)	1.23 (0.30)	0.18 (0.83)			
	CD	0.25 ± 0.03	0.25 ± 0.03	0.25 ± 0.02						
RQ	PD	0.79 ± 0.03	0.80 ± 0.04	0.81 ± 0.05	0.74 (0.40)	3.09 (0.06)	0.99 (0.38)			
	CD	0.78 ± 0.03	0.80 ± 0.03	0.79 ± 0.05						
RMR (kcal/day)*	PD	2105.36 ± 242.63	2110.64 ± 347.73	2181.50 ± 309.44	0.34 (0.56)	0.80 (0.45)	0.14 (0.86)	0.91 (0.35)	0.73 (0.48)	
	CD	2188.73 ± 253.74	2146.82 ± 249.90	2214.09 ± 195.76						
RMR/BM* (kcal/kg/day)	PD	23.68 ± 2.86	24.16 ± 4.37	25.13 ± 3.92	0.88 (0.35)	0.91 (0.40)	0.15 (0.85)			
	CD	25.69 ± 4.05	25.74 ± 6.02	26.47 ± 4.49						
RMR/BSA* (kcal/m^2^/day)	PD	984.00 ± 97.91	993.79 ± 158.94	1031.14 ± 146.03	0.92 (0.34)	0.87 (0.42)	0.13 (0.87)			
	CD	1046.45 ± 113.30	1039.36 ± 169.57	1067.85 ± 112.96						
CHO (g/day)	PD	145.29 ± 55.23	159.64 ± 60.29	189.86 ± 77.20	0.48 (0.50)	3.05 (0.06)	0.68 (0.51)			
	CD	130.55 ± 50.19	162.45 ± 57.49	158.18 ± 80.35						
FAT (g/day)	PD	151.71 ± 34.81	146.00 ± 42.24	139.71 ± 55.01	0.62 (0.44)	1.53 (0.27)	0.68 (0.51)			
	CD	166.82 ± 30.16	148.36 ± 35.68	158.55 ± 45.96						
PRO (g/day)	PD	24.21 ± 3.05	24.21 ± 4.04	24.86 ± 3.81	0.32 (0.57)	0.52 (0.60)	0.13 (0.88)			
	CD	25.27 ± 3.08	24.64 ± 3.05	25.36 ± 2.35						
EE_CHO (kcal/hour)	PD	25.36 ± 9.64	26.43 ± 10.55	32.57 ± 13.21	0.52 (0.48)	2.35 (0.11)	1.10 (0.35)			
	CD	22.36 ± 8.38	27.91 ± 9.87	26.38 ± 13.86						
EE_FAT (kcal/hour)	PD	58.36 ± 13.62	54.50 ± 16.54	51.14 ± 23.37	1.12 (0.30)	1.80 (0.18)	0.66 (0.52)			
	CD	64.36 ± 11.63	57.36 ± 13.78	61.37 ± 18.06						
EE_PRO (kcal/hour)	PD	4.29 ± 0.59	4.29 ± 0.70	4.36 ± 0.61	0.03 (0.86)	2.87 (0.17)	0.85 (0.43)			
	CD	4.27 ± 0.45	4.09 ± 0.51	4.45 ± 0.50						

*PD* Paleo diet, *CD* control diet, *BM* body mass, *BMI* body mass index, *FFM* fat free mass, *VO*_2_ oxygen uptake, *VCO*_2_ carbon dioxide production, *RQ* respiratory quotient, *RMR* resting metabolic rate, *BM* body mass, *BSA* body surface area, *CHO* carbohydrates, *PRO* proteins, *EE* energy expenditure, *SD* standard deviation, * ANCOVA (FFM as covariate), T1 timeline 1, baseline; T2 timeline 2, after 4 weeks; T3 timeline 3, after 8 weeks.

## Discussion

A clear relationship between diet regimen and human resting metabolic rate has not yet been elucidated^33^. Given the current state of research, this is the one of the first study focusing on how a moderate-carbohydrate PD influences RMR in professional athletes. We obtained results demonstrating no effect of the 8-week PD on RMR values in general. It should be noted that throughout the study period, both diets were normoenergetic with significant differences between the macronutrient supply. To date, only one study has been published evaluating the effects of the PD on resting and exercise metabolism in groups of athletes^34^. In Zdzieblik et al.^34^ group differences between paleolithic diet (n = 5) versus a mixed diet (n = 9) were not significant for changes in RER, carbohydrate and fat oxidation rates at rest after 6 weeks of interventions. Similarly, in our research following a PD was not associated changes in RER values under resting conditions. Both diets were of similar energy value comparing energy supply baseline and during the intervention. The Paleo diet is often compared with one of the best researched dietary models, the Mediterranean diet^35–37^. In Daniele et al.^33^, subjects following the Mediterranean diet showed lower carbohydrate and higher lipid intake than those following the vegan diet and, accordingly, a significantly higher BMR and lower RQ. However, it should also be noted that the Mediterranean diet group had a higher average body mass (by approximately 12 kg), which may have contributed, at least in part, to the observed difference in BMR/RMR. Overall, these findings suggest that metabolic profile may be influenced not only by macronutrient intake, but also by differences in body mass. Nevertheless, the data support the notion that the Mediterranean diet, characterized by a higher proportion of proteins and lipids, may be associated with higher BMR and more favorable macronutrient metabolism. Of note, the research group was non-athletes^33^. Green et al.^38^ 14-intermediates to elite competitive lifting athletes consumed an ad libitum usual diet and an ad libitum low-carbohydrate ketogenic diet in random order, each for 3 months in a crossover design, there were no effects in RMR and RQ. Similarly, Che et al.^39^ investigated a short-term fat adaptation followed by carbohydrate restoration diet in well-trained runners. There was no significant difference in RMR and RQ after intervention in experimental and a control high-carbohydrate (HCHO) or between two diet model. When comparing high-carbohydrate (HC) vs. low-carbohydrate (LC) in group of 24 physically active females with correct body mass index (BMI), no significant difference was found for RMR^40^. These results correspond with the results of our own study, which showed no differences between two group in RMR.

The diets applied in our research, both paleo and control involved increased fiber intake (57.35 g (PD) vs 59.75 g (CD) *(p* = *0.488*)), thus hence possibly greater consumption of low glycaemic carbohydrates, which are known to lower postprandial glucose and insulin levels. There is some research indicates that a low-GI meal improved the metabolic profile. Improvements in metabolic responses when consuming low-GI meals may be beneficial to the long-term health of athletes^41^. In our last paper, in the same group there were no differences in carbohydrates (glucose, insulin) and fats (free fatty acids (FFA) and beta-hydroxybutyrate (β-HB), total cholesterol (TC), LDL-C, HDL-C, non-HDL-C, triglycerides (TG) levels) metabolism concentrations during and after the dietary intervention^23^. All indicators stayed stable in correct range during whole experiment. In Karl et al.^42^ research conducted with 91 obese people, moderate-carbohydrate and low-GI diets did not preferentially reduce fat mass, preserve lean mass, or attenuate metabolic adaptation during weight loss compared to high-carbohydrate and high-GI diets^42^.

We observed a significantly higher proportion of energy derived from protein (%) in the PD compared with the rational diet, which may contribute to increased diet-induced thermogenesis and potentially influence RMR. However, there were no changes in utilization of protein in both diets. Targeting macronutrient composition of diets is based on the effect that increasing dietary protein intake might have on metabolic rate or satiety^43,44^. There is also some evidence that the thermogenic effect of digesting greater amounts of dietary protein could account for the apparent greater than expected weight loss during low-carbohydrate ketogenic diet^45^. On the other hand, Mohammadpour et al.^46^ obtained that adherence to diets lower in carbohydrates and higher in fat and protein were associated with higher RMR in Iranian adult, which does not correspond to our results.

What research show there is some evidence, that consuming a low glycaemic diet without energy restriction led to an improved body composition in endurance athletes^47,48^. We obtained no significant changes in body mass, after eight weeks of experiment. Also, FFM did not change after PD what was presented in our last paper ^23^. There is some research in which, weight loss predictably reduces RMR. The rapid and sustained weight and FM loss induced by very low-carbohydrate ketogenic diet (VLCK-diet) in obese subjects did not induce the expected reduction in RMR, probably due to the preservation of lean mass^49^. As we mentioned before, in elite competitive lifting athletes, there were no effects on RMR, as well as in fat mass after consumed an ad libitum usual diet and an ad libitum low-carbohydrate ketogenic diet ^38^. In different research, in trained cyclists with insufficient energy intake during the intense training period decreased in r RMR was observed^50^. So far, our results show that there is no change in RMR,with a nonsignificant slight decrease in BM and maintenance of FFM. The reason for the weight loss with a sustained RMR could be the slightly higher activity-related energy expenditure incurred by the athletes. There is growing interest in the metabolic effects of diet with limited amount of carbohydrate. The potential to preserve RMR especially during non-intentional weight loss is intriguing. We acknowledge that the study has certain limitations, including the relatively short duration of the intervention (8 weeks). Moreover, the present findings apply specifically to the pre-compepition period examined in this study, and results may differ under other training conditions or at different time points. An additional limitation is that protein oxidation/utilization was estimated from indirect calorimetry without measurement of urinary nitrogen. In the present analysis, protein contribution was set at a fixed value of 5%. Since indirect calorimetry alone cannot fully quantify protein oxidation, the related results should be interpreted with caution. We acknowledge that the final sample size was smaller than initially planned. However, further recruitment was not feasible because participants were athletes, and enrolling additional individuals at a later stage would have placed them in different phases of their training cycle, potentially confounding the effects of the intervention (RMR).

## Conclusions

Eight weeks of normoenergetic Paleo diet has no significant effect on RMR in handball players. More research and longer-term interventions are still needed to determine the effect of a moderate-carbohydrate diet on resting metabolism.

## Supplementary Information

Below is the link to the electronic supplementary material.

## Data Availability

The datasets used and/or analyzed during the current study available from the corresponding author on reasonable request.
