# Supplementary material for: Effects of the Paleo diet on resting metabolic rate in handball players
Source: Sci Rep. 2026 Jun 16;16:27617. doi: 10.1038/s41598-026-58374-5 (PMC13538352; doi:10.1038/s41598-026-58374-5)
Supplement: Supplementary file 1 [file 41598_2026_58374_MOESM1_ESM.docx]

## Supplementary Table S1. Example weekly menu of one participant in the PD group.

| **Meal** | **Monday** | **Tuesday** | **Wednesday** | **Thursday** | **Friday** | **Saturday** | **Sunday** |
| --- | --- | --- | --- | --- | --- | --- | --- |
| **Breakfast** | Coconut pancakes with blueberries | Muffin-style frittatas with green vegetables | Chicken salad with sesame and zucchini noodles | Pork loin salad with peas and pickled cucumber | Carrot omelet with coconut cream | Fruit crumble | Roasted pork loin with mango salsa |
| **Lunch** | Chicken roulade with apricots and parsley root purée | Turkey with bell pepper sauce and pea–potato purée | Oriental-style beef with cauliflower rice | Chinese-style chicken with potato–cauliflower purée | Roasted pork loin with sweet potato fries | Roasted rabbit with bacon and peppers | Pork shoulder with fries and broccoli salad |
| **Snack** | Fruit protein smoothie | Mango mousse with coconut flakes | Paleo gingerbread pudding | Paleo apple pie and juice | Pumpkin–banana smoothie | Tomato soup with coconut milk | Green protein smoothie |
| **Dinner** | Cauliflower cream soup with coconut milk | Chicken tartlets | Kohlrabi cream soup with potatoes and chicken | Roasted white vegetable cream soup | Hawaiian salad with mayonnaise | Tuna salad with sun-dried tomatoes | Coconut flour omelet with fruit and nuts |

## Supplementary Table S2. Example weekly menu of one participant in the CD group.

| **Meal** | **Monday** | **Tuesday** | **Wednesday** | **Thursday** | **Friday** | **Saturday** | **Sunday** |
| --- | --- | --- | --- | --- | --- | --- | --- |
| **Breakfast** | Natural yogurt with kiwi and granola | Chickpea salad with egg and basil pesto | Banana and strawberry frittata | Bread with broccoli spread, butter and cheese | Oatmeal with fruit | Sandwiches with pesto and Camembert | Sponge cake squares with feta |
| **Second breakfast** | Fruit smoothie with coconut water | Mexican soup | Sandwiches with peanut butter | Ciabatta with chicken and vegetables | Beetroot cream soup with feta | Mango sunflower seed dessert | Lentil salad with feta |
| **Lunch** | Roasted pork loin with potatoes and beets | Roasted chicken with barley and salad | Chicken in romanesco sauce with whole-grain pasta | Cod in creamy tomato sauce with rice | Oriental-style beef | Turkey with baked polenta | Baked salmon with buckwheat |
| **Snack** | Oat pancakes | Broccoli salad with avocado sauce | Orange buttermilk dessert | Banana bread | Millet brownie with nuts | Salad with dried plums and smoked chicken | Vanilla cottage cheese with fruit |
| **Dinner** | Hawaiian salad with yogurt and bread | Penne with shrimp in tomato sauce | Rye pancakes with lentils and mushrooms | Fish spaghetti in oriental sauce | Chicken tortilla rolls | Root vegetables baked with peanut butter | Coconut risotto with vegetables and parmesan |
