# Supplementary material for: Effects of the Paleo diet on resting metabolic rate in handball players
Source: Sci Rep. 2026 Jun 16;16:27617. doi: 10.1038/s41598-026-58374-5 (PMC13538352; doi:10.1038/s41598-026-58374-5)
Supplement: Supplementary file 2 [file 41598_2026_58374_MOESM2_ESM.docx]

# Table 1a. Example training loads for handball players participating in the study during the pre- competition period - sample microcycle

| *Training component* | *Monday* | *Tuesday* | *Wednesday* | *Thursday* | *Friday* | *Saturday (matches)* |
| --- | --- | --- | --- | --- | --- | --- |
| *General development training* | Gym (90 min): Strength development-lower limbs and chest (high load, low volume; e.g., 4-6 repetitions × 3-4 sets; 80-90% 1RM)  • Barbell back squat (4-5 repetitions × 3-4 sets)  • Barbell bench press (4 repetitions × 3-4 sets)  • Standing calf raise with feet elevated (12 repetitions × 4 sets)  • Reverse lunge (12 repetitions × 3 sets)  • Incline dumbbell chest fly (10 repetitions × 3 sets)  • Biceps exercise | Gym (60 min): Strength development-back and shoulders (high load, low repetitions)  • Conventional deadlift (4-6 repetitions × 4 sets)  • Wide-grip pull-up (6 repetitions × 3 sets)  • Bent-over barbell row (10 repetitions × 3 sets)  • Barbell bench press (6-7 repetitions × 4 sets)  • Standing barbell military press (6 repetitions × 4 sets)  • Standing dumbbell lateral raise (12 repetitions × 3 sets)  • Triceps exercise | Continous long- distance run (60 min) | Gym (60 min): Power development (30-80% 1RM depending on the muscle group; fast movement tempo)  • Hang power clean (5 repetitions × 4 sets)  • Barbell push press (6 repetitions × 3 sets)  • Overhead medicine ball throw performed on a mat (6 repetitions × 3 sets)  • Barbell squat jump (4 repetitions × 3 sets)  • Dynamic barbell bench press (6 repetitions × 4 sets) | X | Selected match (practice match) dates: 6 Oct, 13 Oct, 20 Oct, 9 Nov, 14 Nov, 17 Nov, 1 Dec |
| *Technical-Tactical Training (Handball)* | X | Indoor court (90 min):  • 15 min warm-up:  -running drills (e.g., arm circles, high knees)  -dynamic stretching  -core and stabilization exercises  • 15 min endurance running development:  -interval method with running or movement elements on the court  -runs over distances of 60-80 m  -60 m runs preceded by defensive movement simulation  -effort duration: 15-30 s  -rest duration: 15-40 s  -average: 3 sets of 4-5 interval efforts  • 60 min technical-tactical training  • 10 min stretching | X | Indoor court (90 min):  • 15 min warm-up:  -running drills (e.g., arm circles, high knees)  -dynamic stretching  -core and stabilization exercises  • 20 min speed and power training:  -bounding drills  -hurdle jumps  -change-of-direction sprints  -approximately 10 exercises performed at maximal intensity, with 1-min rest between exercises  -medicine ball throws (overhead, chest pass, rotational): 4 sets × 6 repetitions  • 55 min technical-tactical training  • 10 min stretching | Indoor court (90 min):  • 15 min warm-up:  -running drills (e.g., arm circles, high knees)  -dynamic stretching  -core and stabilization exercises  • 70 min technical-tactical training  • 10 min stretching |  |

*Abbreviation: 1RM, one-repetition maximum.*
